# Clinical and microbiological evaluation of 940-nm diode laser as an adjunct to modified Widman flap for the management of chronic periodontitis: A 6-month randomized split-mouth clinical trial

**DOI:** 10.34172/joddd.2021.023

**Published:** 2021-05-05

**Authors:** Ashish Agarwal, Anugrah Saxena, Shiva Shankar Gummaluri, Bharti Chaudhary, karthikeyan Subramanyam S Sai, Geetika Kumar

**Affiliations:** ^1^Department of Periodontology and Implantology, Institute of Dental Sciences, Bareilly Uttar Pradesh, India

**Keywords:** Chronic periodontitis, Colony-forming units, Diode laser, Periodontal pocket debridement, Surgical flap

## Abstract

**Background.** The present randomized clinical trial aimed to determine the additive clinical and microbiological benefits of diode laser (DL) with modified Widman flap (MWF) to manage chronic periodontitis.

**Methods.** Seventy-two sites in 36 healthy non-smoking patients diagnosed with chronic periodontitis were randomly assigned to the test group (MWF + active DL) or control group (MWF + sham DL). Clinical (probing pocket depth [PPD], clinical attachment level [CAL]) and microbiological (colony-forming units [CFUs]) measurements were recorded at baseline and 6- and 6-month postoperative intervals.

**Results.** Compared to baseline, 6-month results showed significant changes in clinical and microbiological parameters in both groups. However, the intergroup comparison revealed significantly lower PPD (1.90±0.48 mm vs. 2.35±0.41 mm), CAL (4.43±0.57 mm vs. 4.93±0.58 mm), and CFUs for *Porphyromonas gingivalis* (6.32±0.18 vs. 8.88 ±1.88), *Prevotella intermedia* (7.62±1.86 vs. 8.12±1.78), and *Aggregatibacter actinomycetemcomitans* (6.43±1.44 vs. 7.24±1.22) in the test group after six months.

**Conclusion.** Within the limitations, the present study confirmed the useful role of DL with MWF to manage chronic periodontitis.

## Introduction

Microbial biofilm on the root surface within the periodontal pocket is the principal etiologic agent for the disruption of epithelial attachment, loss of supporting periodontal tissues, and inflammatory periodontal disease.^1^ The main goal of periodontal therapy is to remove biofilm and regenerate hard and soft tissue components of the attachment apparatus.^2^

Better accessibility by visual instrumentation is the main inherent advantage of surgical periodontal therapy for treating deep periodontal pockets over the non-surgical periodontal treatment.^3^ However, inadequate removal of invasive tissue microorganisms, epithelial lining, and diseased granulation tissue from the soft tissue wall of the periodontal pocket discourages the attachment of gingival connective tissue to the root surface.^4,5^

In the past few decades, laser-assisted periodontal therapy (LAPT) has emerged as an effective modality for combating gingival and periodontal problems.^6^ LAPT is considered a minimally invasive treatment modality, resulting in less intra- and postoperative discomfort with improved healing and tissue regeneration compared to traditional approaches. Previous literature has shown the impressive safety profile and versatile therapeutic uses of diode laser (DL) in periodontal therapy.^6,7^

Moreover, compelling evidence is available regarding the bactericidal effect of DL for those periodontopathogenic microorganisms that have invaded the soft tissue wall of periodontal pockets.^6^ Diseased periodontal tissues contain melanin, hemoglobin, and chromophores that are the main target of DL energy.^6,8^

Low-level laser therapy (LLLT) emits the wavelength in the visible red and near infra-red (NIR) spectrum (600‒1100 nm). Mitochondrial enzyme cytochrome c oxidase (Cox), the primary photo-acceptor for the red-NIR range, is responsible for the diverse biological cascade observed after laser irradiation. Mitochondria stimulate more ATP production, modulate reactive oxygen species, and activate transcription factors (NF-κB) to induce many gene transcript products responsible for the beneficial effects of LLLT.^9^

Several studies on the adjunctive role of laser beams with scaling and root planing (SRP) have proposed the efficacy of lasers for periodontal diseases.^6^ However, few clinical and microbial studies with conflicting data still maintain a speculative situation regarding the beneficial role of laser therapy with periodontal flap surgery to manage chronic periodontitis. This study aimed to evaluate and compare the effectiveness of the treatment outcomes for laser-assisted periodontal flap surgery with periodontal flap surgery alone in patients with chronic periodontitis by determining the clinical and microbiological parameters. The null hypothesis was that there would be no differences between laser-assisted modified Widman flap (MWF) and MWF alone after six months.

## Methods

**Figure 1:**
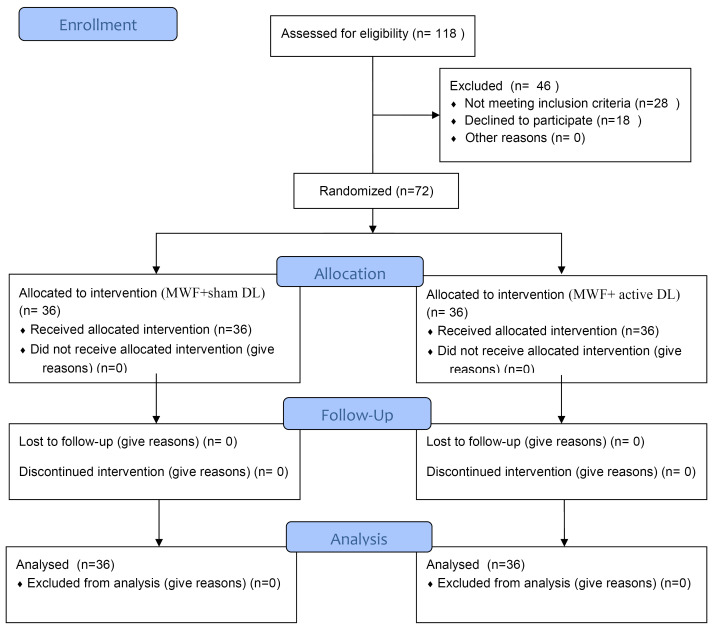
The CONSORT flow diagram of both groups.

This study was designed as a randomized, double-blind, split-mouth, prospective clinical trial with a 6-month follow-up. Seventy-two sites in 36 patients [mean age=49.2±9.04 years (42 males, 30 females)] (Figure 1) with chronic periodontitis (defined by Armitage^1^ in 1999) were included in the study. The study was performed from July 2015 to March 2016. Inclusion criteria consisted of having (1) at least 20 teeth, (2) at least one periodontal pocket in two contralateral quadrants, each with probing pocket depth (PPD) ≥6 mm, clinical attachment level (CAL) ≥6 mm, and plaque index (PI )score < 1^10^ after six weeks of initial non-surgical periodontal therapy. Exclusion criteria consisted of furcation involvement, tooth mobility, systemic disease, smoking, taking any medication, known allergy, pregnancy or lactation, and previous treatment for periodontal reasons.

At the baseline visit, both contralateral sites were randomly assigned to the following control or test group sites by the computer-generated random allocation sequence. The allocation ratio for patients was 1:1.

- Control group: MWF + sham application of a DL
- Test group: MWF + active DL (laser-assisted periodontal therapy)

### Clinical assessment

The primary outcome of this study was CAL; however, PPD, gingival index (GI), PI, sulcus bleeding index (SBI), gingival recession (GR), visual analog scale (VAS), healing index (HI), and colony-forming units (CFUs) were the secondary outcome measures. A single clinician who was blinded to the group assignment recorded all the parameters (PI,^10^ GI,^11^ SBI,^12^ PPD, CAL, GR, and CFU) at baseline (before the treatment), six weeks, and six months after therapy. PPD, CAL, and GR were recorded at six sites per tooth, with a manual UNC-15 periodontal probe (Hu-Friedy, Leinmen, Germany) to the nearest millimeter; the deepest spot of each experimental tooth was defined as the experimental site. A customized acrylic stent with a vertical groove was used for measurements to attain the reproducibility of the probing. For the recording of parameters at different time intervals, the patients were instructed to refrain from any oral hygiene procedure eight hours before the evaluation. Pain assessment by VAS^13^ and HI^14^ were recorded at 1-week postoperative period.

### Microbiological sampling

For subgingival plaque collection, the teeth were isolated with cotton rolls, and a plaque sample was obtained by introducing sterile #40 paper cones into the pocket for 30 seconds. The sample was placed in a vial containing 10 mL of transport media. Furthermore, the samples were placed in Petri dishes containing blood agar under an anaerobic environment (5%‒10% carbon dioxide) at 35‒37ºC for incubation. After 5‒7 days of incubation, the colonies of the selected anaerobic microorganisms (*Porphyromonasgingivalis, Prevotella intermedia*, *Aggregatibacter actinomycetemcomitans)* were identified and counted by an experienced microbiologist. The results were converted into logarithmic values for better understanding and statistical analyses. Plaque samples were collected at baseline and 6-week and 6-month postoperative intervals.

Teeth with pockets measuring ≥5 mm in depth in the same quadrant as the study teeth were surgically treated. The surgical treatment was performed by a single clinician unaware of the study protocol.

### Treatment phase

**Figure 2:**
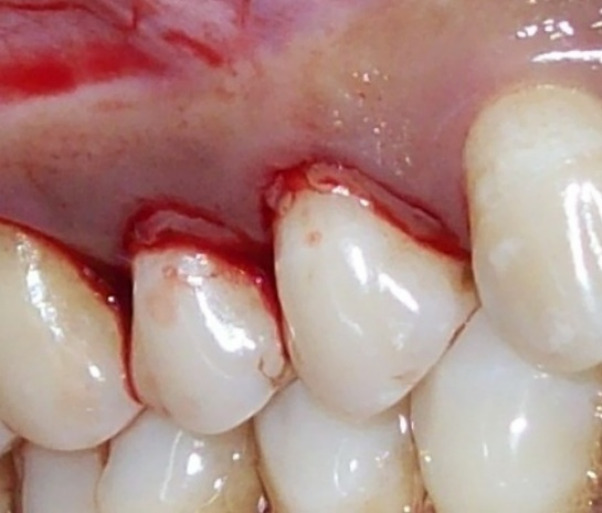
Incision; the control group.

**Figure 3:**
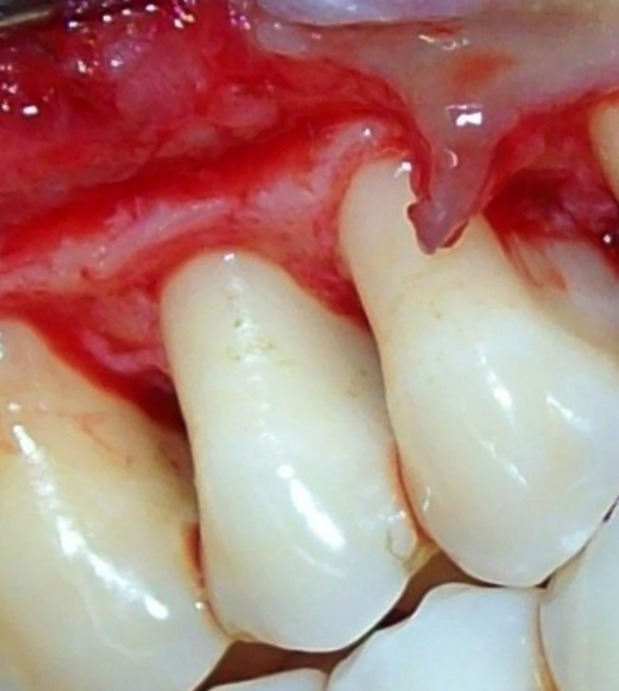
Flap reflection and debridement; the control group.

**Figure 4:**
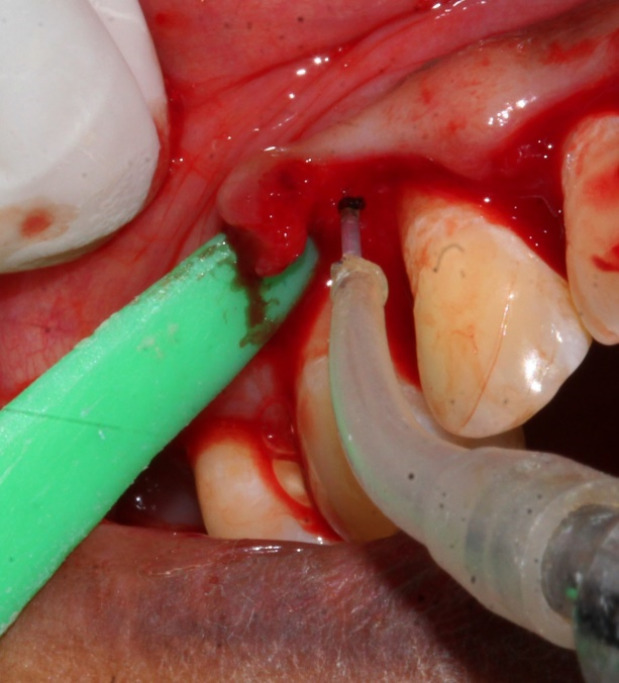
Test group de-epithelization with diode laser.

Control sites (Figures 2 and 3) were treated with MWF and sham application of DL therapy, whereas test sites underwent MWF and active DL application. In all the sites, the MWF was followed by SRP and elimination of granulation tissue using hand and power-driven instruments. Before each irradiation episode, a power meter (Fieldmaster, Coherent, Alburn, USA) was used, which allowed the adjustment and standardization of the amount of energy used. For test sites, a DL (wavelength = 940 nm, power = 1 W, tip diameter = 400 µm, power density = 796 W/cm^2^) was used in continuous mode to remove visible epithelium from the undersurface of the flap. The DL irradiation was carried out at a 45º angle to the soft tissue for 10 seconds from the coronal to apical aspect in parallel paths, followed by 30 seconds of interruption. The charing layer, which was produced due to laser application, was removed with moist gauze. A second laser application (LLLT) was carried out on all the surfaces of the ﬂap (under and outer, exposed bone, and exposed root structures) in continuous mode at 0.1 W, adding up to a total dose of 4 J/cm^2^ per surface (Figure 4). The flaps were sutured with 3-0 black silk sutures. Ibuprofen with a 200-mg dose was prescribed every eight hours for five days for pain control. Furthermore, patients were requested to avoid brushing in the treated area for two weeks. They resumed full oral hygiene and function after two weeks.

### Statistical analysis

The results were averaged (mean ± SD) for each clinical and radiographic parameter at baseline, 6-week, and 6-month intervals. The intragroup comparison was made using the Friedman test, where *P* < 0.05 was considered statistically significant. Post hoc analysis was carried out using the Wilcoxon signed-rank test, and *P* < 0.004 and *P* < 0.0002 were considered statistically significant (after applying Bonferroni correction) for periodontal and microbial parameters, respectively. The intergroup comparison was carried out using the Mann-Whitney U test, where *P* value < 0.05 was considered statistically significant.

Power analysis was performed before the study was initiated. To achieve a 90% power and detect mean differences of 1 mm for CAL between the groups, 25 sites per group were required. The mean intraexaminer standard deviation of differences in repeated PPD and CAL measurements was obtained using single passes of measurements with a periodontal probe (correlation coefﬁcients between duplicate measurements; r=0.95).

## Results

Table 1 presents the demographic data. Both groups showed significant changes in PPD (68.13% and 69.9%, respectively), CAL (44.2% and 47.3%, respectively), and CFUs of anaerobic bacteria after six months compared to the baseline. However, PI, GI, and BOP exhibited non-significant changes after six months, indicating that the patients in both groups showed strict compliance for oral hygiene throughout the study. On intergroup comparison, mean PPD and CAL were significantly lower in the test group than the control group at six weeks and six months. However, GI, PI, BOP, and GR exhibited non-significant differences in both groups at all the time intervals (Tables 2 and 3).

**Table 1:** Demographical data of the study

	**MWF + active DL (36 sites in 18 patients)**	**MWF + sham DL (36 sites in 18 patients)**
Male (42)	25	17
Female (30)	11	19
**Teeth treated**		
Mandibular premolars (7)	4	3
Maxillary premolars (18)	10	8
Mandibular molars (22)	10	12
Maxillary molars (25)	12	13

**Table 2:** Intragroup and intergroup comparisons of clinical parameters

**Parameters**		**Baseline**	**6 weeks**	**6 months**	***P*** **value** ^a^	**Mean change (%)**
GI	Control	0.61±0.17	0.63±0.22	0.71±0.17	0.28	14.1
	Test	0.60±0.20	0.62±0.19	0.65±0.18	0.88	7.7
	*P* value^b^	0.99	0.88	0.20	-	-
PI	Control	0.62±0.17	0.64±0.16	0.65±0.18	0.15	4.6
	Test	0.60±0.19	0.61±0.19	0.63±0.16	0.26	4.8
	*P* value^b^	0.78	0.55	0.54	-	-
BOP	Control	0.90±0.21	0.88±0.22	0.82±0.32	0.12	8.9
	Test	0.88±0.28	0.83±0.29	0.80±0.33	0.10	9.1
	*P* value^b^	0.18	0.15	0.17	-	-
PPD	Control	6.73±0.93	2.30±0.41	2.35±0.41	0.00*	68.13
	Test	6.33±1.03	2.00±0.61	1.90±0.48	0.00*	69.9
	*P* value^b^	0.81	0.02*	0.01*	-	-
CAL	Control	8.83±0.70	4.97±0.61	4.93±0.58	0.00*	44.2
	Test	8.70±0.62	4.47±0.57	4.43±0.57	0.00*	47.3
	*P* value^b^	0.25	0.00*	0.00*	-	-
GR	Control	2.46±0.65	2.67±0.72	2.58±0.58	0.14	4.9
	Test	2.37±0.74	2.47±0.71	2.53±0.68	0.08	6.8
	*P* value^b^	0.18	0.06	0.66	-	-

^a^ *P* value (Friedman test) over time, intragroup comparison, *P < 0.05. ^b^ *P* value (Mann-Whitney U test) intergroup comparison, *P < 0.05.

**Table 3:** Intragroup comparisons of PPD and CAL at different time periods

	**PPD**		**CAL**	
	**Control group**	**Test group**	**Control group**	**Test group**
Baseline – 6 weeks	0.00*	0.00*	0.00*	0.00*
Baseline – 6 months	0.00*	0.00*	0.00*	0.00*
6 weeks – 6 months	0.059	0.007	0.317	0.317

Wilcoxon signed ranks test, *P < 0.004

After one week, the VAS score in the test group (2.02±0.55) was significantly better than the control group (2.86±0.83). HI exhibited a significant, beneficial shift in the test group (4.12±0.51) compared to the control group (4.98±0.49) after one week (Table 4).

**Table 4:** Intergroup comparisons of VAS and HI

**(One week postoperatively)**	**Control group**	**Test group**	***P*** ** value**
VAS	2.86±0.83	2.02±0.55	0.02*
HI	4.98±0.49	4.12±0.51	0.03*

Mann-Whitney U test, *P < 0.05

Concerning intergroup microbial analysis and comparison, the test group exhibited significantly fewer CFUs of anaerobic microorganisms than the control group at 6-week and 6-month postoperative intervals (Tables 5 and 6).

**Table 5:** Intragroup and intergroup comparisons of microbiological parameters [CFUs of bacteria (log)]

		**Baseline (mean ± SD)**	**6 weeks (mean ± SD)**	**6 months (mean ± SD)**	***P*** **value** ^a^	**Mean change (%)**
*P. gingivalis*	Control	10.00±1.55	8.63±1.64	8.88±1.88	0.00*	11.2
	Test	10.52±1.82	6.22±1.72	6.32±0.18	0.00*	40
	*P* value^b^	0.99	0.00*	0.00*	-	-
*P. intermedia*	Control	9.44±2.32	8.02±1.98	8.12±1.78	0.00*	14
	Test	9.37±2.11	7.22±1.19	7.62±1.86	0.00*	18.6
	*P* value^b^	0.78	0.00*	0.00*		
*actinomycetemcomitans*	Control	8.04±1.88	7.12±1.42	7.24±1.22	0.00*	9.9
	Test	8.14±1.67	6.10±1.22	6.43±1.44	0.00*	21
	*P* value^b^	0.12	0.00*	0.00*		

^a^ *P* value (Friedman test) over time, intragroup comparison, *P < 0.05. ^b^ *P* value (Mann-Whitney U test) intergroup comparison, *P < 0.05.

**Table 6:** Intragroup comparisons of microbial CFUs at different time intervals

**Comparison**	***P. *** ***gingivalis***		***P. intermedia***		***A. *** ***actinomycetemcomitans***	
	**Control**	**Test**	**Control**	**Test**	**Control**	**Test**
Baseline – 6 weeks	0.001*	0.000*	0.001*	0.000*	0.001*	0.000*
Baseline – 6 months	0.001*	0.000*	0.001*	0.000*	0.001*	0.000*
6 weeks – 6 months	0.005	0.005	0.008	0.005	0.009	0.003

Wilcoxon signed-ranks test, *P < 0.002

## Discussion

This study was designed as a split-mouth investigation that had the inherent advantage of eliminating inter-individual variations or patient-specific characteristics from the treatment effect estimates. The results showed significant differences in clinical and microbiological parameters in both groups six months postoperatively. During the intergroup comparison, the test group (MWF + active DS) exhibited significantly more benefits than the control group (MWF + sham DL) for managing chronic periodontitis. No patient reported any complication and impaired tissue response after treatment and during the follow-up.

Previous literature does not provide a firm viewpoint regarding the role of laser-assisted periodontal surgery. A recent split-mouth randomized study is consistent with our findings concerning more significant clinical and microbiological improvements for periodontal flap with DL than periodontal flap alone.^15^ However, a smaller sample size and involvement of quantitative real-time polymerase chain reaction instead of CFUs were the differences from our study. Another study also showed significantly more gain in PPD and CAL with the laser-assisted periodontal flap than periodontal flap alone, with more significant benefits, which might be due to greater initial PPD in that study.^16^ Our results are also consistent with a previous clinical trial by Ozcelik et al,^17^ who reported superior outcomes in PPD, CAL, GR, VAS, and gingival swelling with enamel matrix derivative + LLLT than enamel matrix derivative alone.

According to Gokhale et al,^18^ more significant reduction in microbial CFUs was found in the test group (DL + flap surgery) than the control group (flap surgery alone), while the clinical parameters were similar in the two groups. A recent study reported no additional advantages for DL with conventional access flap debridement than periodontal flap alone.^19^ Moreover, Lobo and Pol^20^ reported more gingival inflammation reduction in DL associated with flap surgery than flap surgery alone; however, their study did not show any difference in PPD and CAL in the intergroup comparison.

Sanz-Moliner et al^9^ and Heidari et al^21^ assessed the use of DL with periodontal flap surgery for chronic periodontitis and observed the additional benefits for tissue response and pain perception, consistent with our study.

DL has certain inherent advantages due to its smaller size, low cost, and ease of handling. It has a wavelength range of about 800–980 nm, which is appropriately absorbed by hemoglobin and other pigments, thus speciﬁcally targeting the pigmented bacteria and diseased granulation tissue, 3et al^29^ histologically examined the in vivo effects of DL irradiation on the root surface and verified its safety profile in terms of thermal deterioration of cementum.

A probable explanation for the observed heterogeneity for laser studies might be variations in laser types, application intervals, duration, power setting, mode, population characteristics, surgical procedures, parameter severity, and duration of the study. Before generalizing the findings of any clinical trial, it should be tested several times by different scientists/clinicians on different population pools to prevent subjective turmoil and prove the predictability and reliability of the results.^30^

## Conclusion

The DL can be used as an adjunct to the MWF for significant added clinical and microbiological benefits compared to the MWF alone to manage chronic periodontitis.

## Authors’ Contributions

AA and AS were responsible for the concept or design of the work, data acquisition or analysis, interpretation, revising, and final drafting of the work. GSS was responsible for data acquisition or analysis, editing, and final drafting of work. CB, SSSK, and GK were responsible for the analysis and revision of work.

## Funding

The** s**tudy was self-funded by authors themselves and did not receive any funding from any agency.

## Competing Interests

The authors declare no competing interests with regards to the authorship and/or publication of this article.

## Ethics Approval

The study was approved by the institutional review board under the code IDS/ETHCC/15/07The study was conducted following the Helsinki declaration of 1975 as revised in 2000, and all the participants signed informed consent forms.
